# Ecotoxicological assessment of cigarette butts on morphology and photosynthetic potential of *Azolla pinnata*

**DOI:** 10.1186/s12870-024-04991-z

**Published:** 2024-04-18

**Authors:** Garishma Shah, Upma Bhatt, Hanwant Singh, Deepak Kumar, Jyotshana Sharma, Reto J Strasser, Vineet Soni

**Affiliations:** 1 Plant Bioenergetics and Biochemistry Lab, Mohanlal Sukhadia University, Udaipur, Rajasthan India 313001; 2 Plant Bioenergetics Laboratory, University of Geneva, Jussy, 1254 Geneva, Switzerland

**Keywords:** Cigarette butts, Chlorophyll fluorescence, Energy fluxes, Quantum yield, Performance index, Photosynthesis

## Abstract

Cigarette butts (CBs) have become the most ubiquitous form of anthropogenic litter globally. CBs contain various hazardous chemicals that persist in the environment for longer period. These substances are susceptible to leaching into the environment through waterways. The recent study was aimed to evaluate the effects of disposed CBs on the growth and development of *Azolla pinnata*, an aquatic plant. It was found that after a span of 6 days, the root length, surface area, number of fronds, and photosynthetic efficacy of plant were considerably diminished on the exposure of CBs (concentrations 0 to 40). The exposure of CBs led to a decrease in the F_M_, F_V_/F_0_, and φP_0_, in contrast, the φD_0_ increased in response to CBs concentration. Moreover, ABS/CSm, TR_0_/CSm, and ET_0_/CSm displayed a negative correlation with CB-induced chemical stress. The performance indices were also decreased (*p*-value ≤ 0.05) at the highest concentration of CBs. LD_50_ and LD_90_ represent the lethal dose, obtained value for LD_50_ is 20.30 CBs and LD_90_ is 35.26 CBs through probit analysis. Our results demonstrate that the CBs cause irreversible damage of photosynthetic machinery in plants and also reflect the efficacy of chlorophyll *a* fluorescence analysis and JIP test for assessing the toxicity of CBs in plants.

## Introduction

Cigarette butts (CBs) have unfortunately maintained their position as the most prevalent form of litter item globally since the 1990s [1–3]. Approximately, 4.5 trillion CBs are discarded into the atmosphere each year [2] and in which 76 % to 84 % of smokers discard their CBs directly on the land instead of disposing them in a bin [4, 5]. This contribute 22-46 % visible waste in metropolitan areas worldwide [4–6].. An average density of finding CBs in an area is 2.7 CBs m^-2^ highest is 47 CBs m^-2^ in Berlin ([7] . Most of CBs consist with a filter to ease the direct effect of CBs chemicals to smokers. This filter is fabricate by 12,000 cellulose acetate fibers and can persist in the surroundings for an unknown period [8, 9] (. When discarded, CBs often contain un-smoked tobacco and various toxicants like nicotine, polycyclic aromatic hydrocarbons (PAHs) [7], formaldehyde, BTEX [10], acrolein, benzene derivatives, polycyclic aromatic amines [11, 12] , poisonous gases, tar, and heavy metals, [13]. Some cigarettes also contain flavorings agents mostly in menthol based cigarettes, such as 5-methyl-2-(propan-2-yl)cyclohexan-1-ol [14, 15]. Improper disposal of CBs on land leads to the toxicants becoming accessible in plants and animals [16, 17]. This is due to the chemicals being easily washed into the environment through waterways and runoff when exposed to atmospheric moisture and rain [7, 18] . . The detrimental effects of cigarettes on human health are widely documented in the literatures [15]. Still, there is a lack of understanding about their environmental impacts.

In aquatic habitats, these toxic compounds can leach, causing harm to marine and fresh water organisms. Evidence suggests that aquatic organisms, such as *Ceriodaphnia dubia* [18], *Pimephales promelas* [13], bacteria, *Hediste diversicolor* [19], gastropods [20], and *Atherinops affinis* [13], are vulnerable to the lethal properties of these compounds. Interestingly, even un-smoked CBs were found to be lethal to freshwater fishes and few marine fishes, also in the some species of fresh water fishes have unconscious nervous system on the exposure of high CBs concentration [13]. The accessibility of toxic substances to organisms is influenced by their leaching capacity [21]. Nicotine, for instance, is highly soluble in water, especially under alkaline conditions, and can percolate from CBs over time [22]. Several plant products, including food crops, teas, and spices, have been found to contain nicotine, which can be taken up by plants from tobacco smoke or soil littered with commercial tobacco [22]. Recent short-term experimental exposure to leachate from smoked cigarette filters showed mutagenic, genotoxic, and cytotoxic effect in onion plants [23]. However, the effects of littered CBs on plant germination, growth, and chlorophyll amount, photosynthesis are not well understood [24].

*Azolla* (Lam.), commonly mosquito fern, is a fast-growing, free-floating aquatic plant that has been widely used in aquatic biology research due to its rapid growth. This plant has numerous applications, including its use as a livestock feed, human food, bio-fertilizer, and a bio-fuel [25]. Among the diverse species of mosquito fern*, Azolla pinnata* has been one of the most frequently used in experimental works because of its large abundance in rice fields, reservoirs and polluted ponds. Several studies found to asses toxicity of Pb [26], Cd, Cr [27], rhodamine B dye [28] using *A. pinnata* plant. Our study focused to assess the impact of CBs exposure on the physiology and morphology of *A. pinnata*, with a particular emphasis on photosynthetic efficiency using various parameters. Chlorophyll* a* fluorescence (ChlF) is a widely used non-persisting technique to sense plant stress conditions, and it is often combined with other physiological and chemical variables [29, 30]. ChlF describes the natural procedure by which absorbed energy is not utilized for photosynthesis which is dissipated as heat or re-emitted, providing a quantitative assessment of oxygenic photosynthesis [31]. CBs exposure disrupts photosynthesis by chemically interacting with proteins and increasing reactive oxygen species generation [32, 33]. This study provide a wide information about the impact of CBs on plants only some basics elementary studies has been done and mentioned in the Table 1 [34]. The study provides the impact of severity of CBs on plants growth, metabolism and physiology. The hypothesis that on the continuous and long exposure of CBs in the atmosphere produces ill effect in plant measure with the help of physiological and morphological observation.

**Table 1:** Previous elementary work on exposure of CBs on different plants

S.N.	Plant species	Effect of Chemical released by Cigarette Butts	
1	*Glycine max*	Reduction in vessels diameter, ylem and phloem in vascular bundles	(Weryszko-Chmielewska & Chwil, 2005)
2	*Pisum sativum*	Reduction observed in biomass, root/shoot ratio and leaf area	(Çimrin et al., 2007)
3	*Cicer arietinum*	Reduction in number of leaves	(Das et al., 2012)
4	*Arabidopsis thaliana*	Reduction in lipid peroxidation, reactive oxygen species, GSH and ascorbate which lead to cell death	(Zhao & Yi, 2014)
5	*Lolium perenne*	Initial growth being stunted	(Green et al., 2019) [35]
6	*Brasicca napus*	Reduce root length due to the exposure of PAHs	(Zhao & Yi, 2014)
7	*Gossypium hirsutum* and *Catharanthus roseus*	Low photo synthesis due to reduction in chlorophyll a and b and plant is in stress	(Iori et al., 2017)
8	*Triticum aestivum*	Seedling shows genotoxic effect, growth retardation and cellular damage observed	(Abbas et al., 2017)
9	*Allium cepa*	Cytotoxic, genotoxic and mutagenic effect	(Montalvão et al., 2019) [23]
10	*Suaeda salsa*	High concentration produce adverse effect on the growth	(Xu et al., 2020)

## Materials and methods

### Plant material and CBs collection

*Azolla pinnata* plants were obtained from a pond located at the botanical garden of the Department of Botany, Mohanlal Sukhadia University, Udaipur, India (coordinates 24° 34'54" N and 73° 42'40" E). The plant specimen was identified by Dr. Vineet Soni on the basis of characteristic like leaves are tiny, lacy-looking, and closely overlapping, leaves can be green or rusty red and short, branched, floating stem, bearing roots which hang down in the water. The leaves are alternately arranged, each consisting of a thick aerial dorsal lobe containing green chlorophyll and a slightly larger thin, colourless, floating ventral lobe and deposited in the herbarium of the Department of Botany, MLS University, Udaipur, India, with accession number-MLSU/BOT/00227896. CBs were collected randomly from roadsides, parks, and smoking areas. The collected CBs were of similar length but from different brands. For the experiment, surrounding trapping paper of CBs was removed by the help of forceps and scissors. The CBs were dried overnight at 40 °C and then used to prepare concentrated leachate of different concentrations 10CBs L^-1^, 20 CBs L^-1^, 30 CBs L^-1^, 40 CBs L^-1^ [36]. The experiment was repeated several time in order to screen the concentration of CBs. On the basis of these primary screenings, the best and suitable concentration of CBs in number per liter was selected for further studies. The leachate samples were subjected to an ecotoxicological test to assess their potential environmental impact.

### Experimental design

After being collected, *A. pinnata* plants were transferred to a water tank amended with a solution called MPK solution 1 g L^-1^ (Magnesium chloride, rock phosphate, and potassium salt in 1:2:1 w/w/w ratio) every 15 days for propagation at the Plant Bioenergetic and Biochemistry Laboratory at MLS University in Udaipur, India. For the experiments, healthy 7-day-old plants were moved into 1 L glass containers filled with a nutrient solution containing 1000 µM (NH_4_)_2_SO_4_, 1000 µM Ca(NO_3_)_2_, 500 µM K_2_SO_4_, 500 µM MgSO_4_, 250 µM KH_2_PO_4_, 10 µM Fe-EDTA, 10 µM H_3_BO_3_, 0.5 µM MnCl_2_, 0.5 µM ZnSO_4_, 0.1 µM CuSO_4_, and 0.1 µM (NH_4_)_6_Mo_7_O_24_ (pH of the nutrient solution was adjusted to 6.5 ± 0.2 applying NaOH or H_2_SO_4_) and placed in the plant growth chamber. All chemicals are purchased from Sigma-Aldrich, Udaipur, India. The chamber was maintained at specific environmental conditions including a 16-hour light and 8-hour dark photoperiod with an intensity of 50 µmol photons m^−2^ s^−1^, temperature is 25-28 °C, and 70-75 % relative humidity. After one week acclimatization period, plants (≈ 50 healthy fronds) were relocated to 1 L glass containers (having 20 cm diameter and 10 cm height) with diverse concentrations of CBs (10, 20, 30, 40 CBs) mixed with the same nutrient solution and allowed to grow for 6 days. Each treatment had three independent replicates (*N*=3) and an equal amount of plant biomass.

### Measurement of morphological parameters

Determination of Average Fronds area (AFA), Average fronds number (AFN) and Average root length (ARL), specific morphological variability in *A. pinnata* exposed to CBs, the Fiji-Image J software (an open-source software used for advanced processing and scientific analyses of images, https:// imagej.net/Fiji) was used. Plant images were captured every interval of 3 days for up to 6 days duration using a DSLR camera (Nikon D7500, Resolution 20.9MP and distance from object is 70 cm) under dispersed light conditions.

### Measurement of chlorophyll a fluorescence

The plant efficiency analyzer Handy PEA fluorimeter, manufactured by Hansatech Instruments Ltd. England, was used to measure ChlF. Prior to measurement; fronds were subjected to a dark adaptation period of 50-60 minutes at 26 °C. The Biolyzer v.3.0.6 software, developed by the Laboratory of Bioenergetics at the University of Geneva, Switzerland, was used to analyze the ChlF signals. To ensure the accuracy of the results, the experiments were performed in six replicates and repeated thrice times. The JIP-test method was utilized to calculate various phenomenological and biophysical parameters that quantify the behaviors of both the photosystem I (PSI) and photosystem II (PSII). The polyphasic ChlF rise, also known as the OJIP curve, provided valuable information about photosynthetic fluxes, and numerous parameters were derived from it [37, 38] \ Table 2 presents the definitions, formulas, and abbreviations for the JIP-test parameters utilized in the current study.

**Table 2:** The JIP-test parameters, along with their respective abbreviations, formulas, and definitions, are presented

BASIC PARAMETERS CALCULATED FROM THE EXTRACTED DATA	
FO $\cong$ F50µsor $\cong$ F20µs	fluorescence when all PSIIRCs are open ($\cong$ to the minimal reliable recorded fluorescence) [39]
T_FM_=tF_MAX_, t for F_M_	Time (in ms) to reach maximal fluorescence Fm [39]
FM(=FP)	maximal fluorescence, when all PSIIRCs are closed (=FP when the actinic light intensity is above 500 µmol (photon) m^-2^ s^1^ and provided that all RCs are active as QA-reducing) [39]
FV $\equiv$ FM – FO	maximalvariablefluorescence [39]
SM $\equiv$ Area/(FM – FO)=Area/FV	NormalisedArea to Fm [39]
N =SM $\times$ (MO/VJ)	Turnovernumber(expresseshowmanytimesQAisreducedinthetimeintervalfrom 0 to tF_M_) [39]
V_J_ = (F_J_ – FO)/(F_M_ – FO)	Relative variable fluorescence at t = 2 ms [39]
V_I_ = (F_I_ – Fo)/(F_M_ - Fo)	Relative variable fluorescence at t = 30 ms [39]
BIOPHYSICAL PARAMETERS DERIVED FROM THE BASIC PARAMETERS	
* DeexcitationrateconstantsofPSIIantenna*	
kN_=_(ABS) $\times$ kF $\times$ (1/FM)	Nonphotochemical deexcitation rate constant (ABS: absorption flux - see below; kF: rate constant for fluorescence emission) [39]
kP_=_(ABS) $\times$ kF $\times$ (1/FO – 1/FM)=kN $\times$ (FV/FO)	Photochemical deexcitation rate constant [39]
* Specific energy fluxes (perRC: QA-reducing PSII reactioncentre),inms-*^*1*^	
ABS/RC_=_ MO $\times$ (1/VJ) $\times$ (1/φPo)	Absorption flux (exciting PSII antenna Chl a molecules) per RC (also used as a unit-less measure of PSII apparent antenna size) [39]
TRO/RC _=_MO $\times$ (1/VJ)	Trapped energy flux (leading to Q_A_ reduction), per RC [39]
ETO/RC _=_MO $\times$ (1/VJ) - (1-VJ)	Electron transport flux (further than Q_A_^-^), per RC [39]
DIo/RC _=_ ABS/RC– TRo/RC	Dissipated energy flux per RC (at t = 0) [39]
* Phenomenologicalenergyfluxes(perCS:QA-reducingPSIIcrosssection),inms-*^*1*^	
TRO / CS_M=_(Fv/F_M_) (ABS/CS_M_)	Trapped energy flux (leading to Q_A_ reduction) per RC (Tsimilli-Michael, 2020 [39])
ETO / CS_M=_(Fv/F_M_) (1 - V_J_) (ABS/CS_M_)	Electron transport flux (further than Q_A_^-^) per RC (Tsimilli-Michael, 2020 [39])
DIO / CS_M=_(ABS/CSO) - (TRO/CSm)	Total energy dissipated per reaction center (RC) (Tsimilli-Michael, 2020 [39])
ABS / CS_M=_≈ *F*o	Absorbed photon flux per excited PSII cross section at time zero [39]
* Quantumyieldsandefficiencies*	
φPo=TR0/ABS=[1 - (FO/FM)]	Maximum quantum yield for primary photochemistry [39, 40]
φEo=ET0/ABS=[1- (FO/FM)] (1-VJ)	Quantum yield for electron transport (ET) [41]
ψEo=ET0/TR0=(1-VJ)	Efficiency/probability that an electron moves further than Q_A_^-^ [41]
ϕDo= Fo/Fm	Quantum yield (at t = 0) of energy dissipation [41]
* Performance indexes*	
${PI}_{ABS}=\frac{1-({F}_{O}/{F}_{m})}{{M}_{O}/{V}_{j}}\times \frac{{F}_{m}/{F}_{o}}{{F}_{O}}\times \frac{1-{V}_{j}}{{V}_{j}}$	Performance index for energy conservation from photons absorbed by PSII until the reduction of intersystem electron acceptors [39, 41]
${PI}_{CS}=\frac{ABS}{CS} \times \frac{1-({F}_{O}/{F}_{m})}{{M}_{O}/{V}_{j}}\times \frac{{F}_{m}/{F}_{o}}{{F}_{O}}\times \frac{1-{V}_{j}}{{V}_{j}}$	Performance index on cross section basis [39, 41]

### Statistical analysis

In this study, a statistical analysis was performed to evaluate the significance of measurements using ANOVA conducted by a Tukey HSD test (*p* = 0.05) with the use of SPSS software (version 22.0). The figures presented only include measurements that had a significant value of *p* ≤ 0.05. To create an unbiased color code, the values were normalized and scaled between 1 and 100, with a color scheme of red indicating high values (100%), yellow indicating medium values (50%), and green indicating low values (1%) used to generate the heat map. The correlation grid was designed using Microsoft Excel and Canva software. Additionally, a principal component analysis (PCA) was performed using Origin Pro 2018 software to identify any patterns and variations in the experimental data through eigenvalue decomposition of a data correlation matrix. The ChlF parameter was selected for the PCA analysis, which helped in identifying the variables that showed the highest fluctuations. The results showed that dimension 2 (PC 2) accounted for 74.09 % of the maximum variability, while dimension 1 (PC 1) accounted for 19.63 %. The correlation between the parameters was analyzed using a grid correlation matrix and expressed using a color code between +1 and -1 by using python software [42, 43]. Probit Analysis, with the use of SPSS (22.0), was conducted to determine the lethal dose (LD_50_ and LD_90_), while a Chi-square test was employed to compare the mortality ratios between experimental and control groups at varying concentrations.

## Results:

The growth and productivity of *A. pinnata* was significantly affected by the occurrence of CBs, which caused modulation of the plant's photosynthetic process. To investigate this phenomenon, the current study explored the impact of CBs on various parameters of *A. pinnata*, including morphological parameters, chlorophyll fluorescence, specific energy fluxes, phenomenological energy fluxes, and performance indexes.

### Morphological parameters

**Fig. 1:**
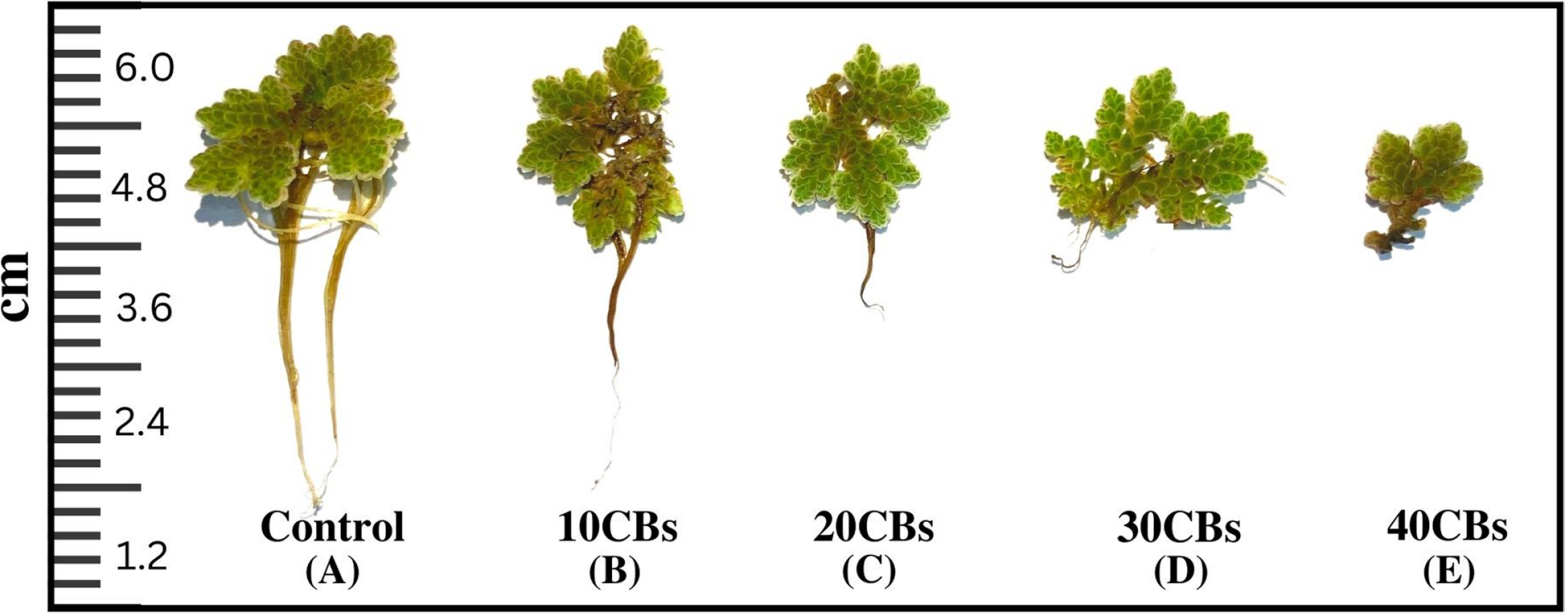
Morphological changes in the *A. pinnata* during exposure of differential concentration of CBs (**A**) 0 CBs, (**B**) 10 CBs, (**C**) 20 CBs, (**D**) 30 CBs, (**E**) 40 CBs

**Fig. 2:**
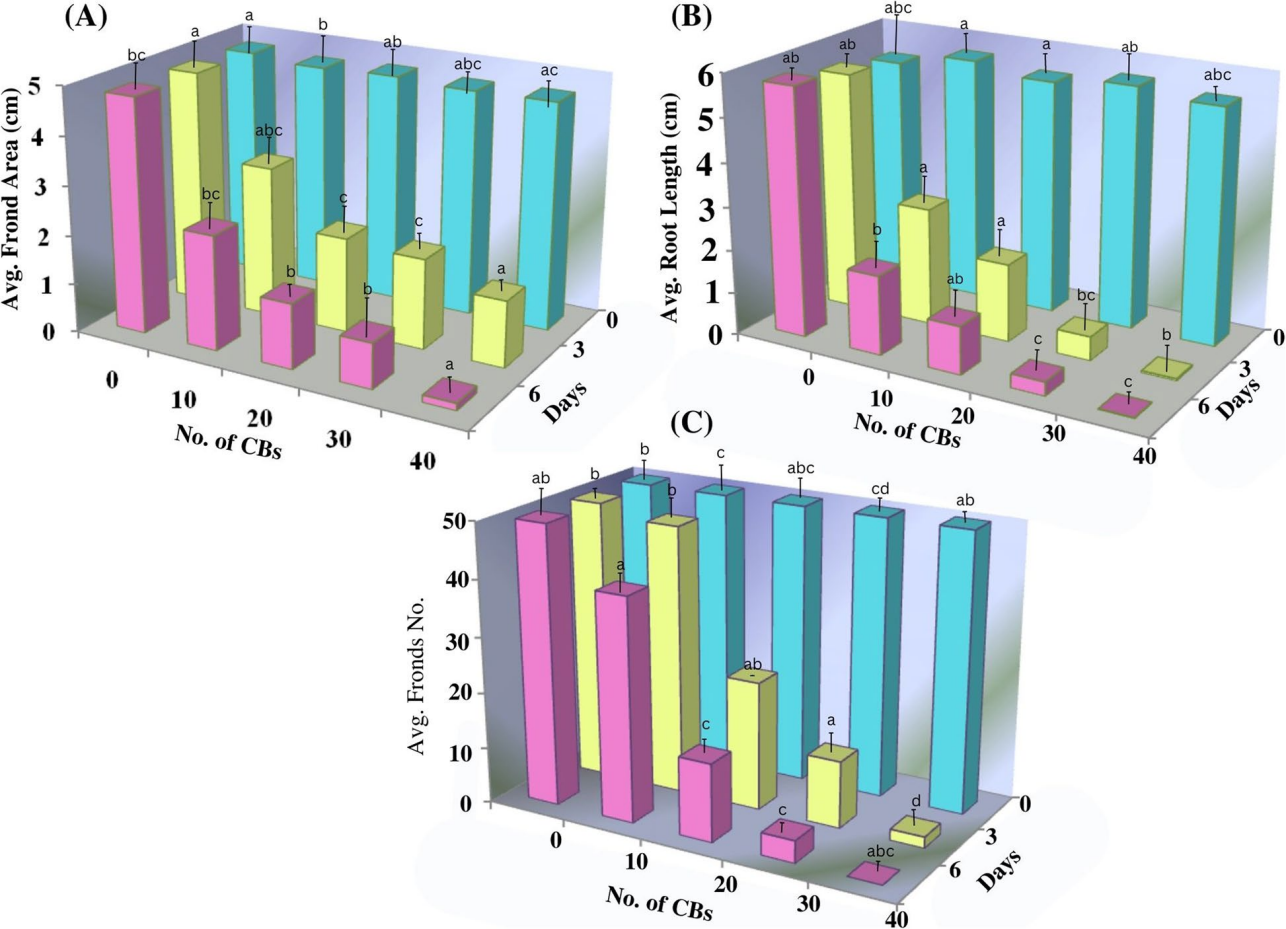
3D bar graph representing the (**A**) change in average surface area (ASA), (**B**) change in average root length (ARL), (**C**) change in average fronds number (AFN) in *A. pinnata* on the exposure of differential concentration of CBs (0 to 40 CBs)

The study observed a significant decrease in the average frond area (AFA) of *A. pinnata* with increasing concentrations of CBs, with a standard deviation of ± 0.1. The reduction in AFA was found to be continuous as the concentration of CBs increased. By the 6th day of the experiment, the AFA was decreased by 3% compared to the control plants. Additionally, the root length (ARL) of *A. pinnata* was found to be reduced from 5-6 cm in control plants to 0.25 cm in plants exposed to CBs. Differential changes in AFA and ARL are present in Fig. 1 (A to E).The increase in CBs concentration was also found to have a negative impact on plant mortality, with a reduction in the number of fronds (AFN) observed. At the end of the experiment, average two plants were found to be alive in the 40 CBs concentration condition shown in Fig. 2. These findings suggest that exposure to CBs has a significant negative impact on the growth and survival of *A. pinnata*.

### Biochemical parameters

Throughout the experiment the chl *a* and *b* concentration were significantly decrease with the increasing concentration of CBs. Highest value of chl *a* and *b* were observed in the control system is 0.625 ± 0.022 and 0.443 ± 0.026, respectively. The lowest value is approximate half value from control as demonstrate in the Table 3.

**Table 3:** Mean values of the Chlorophyll *a* and chlorophyll *b* content measured in *A.pinnata* after exposure of various concentrations of CBs

**S.No.**	**Treatment**	**Chl *a* (mg g**^**-1**^** FW)**	**Chl *b* (mg g**^**-1**^** FW)**
1.	Control	0.625 ± 0.022^a^	0.443 ± 0.026^a^
2.	10 CBs	0.557 ± 0.003^a^	0.425 ± 0.002^a^
3.	20 CBs	0.412 ± 0.018^b^	0.339 ± 0.010^ab^
4.	30 CBs	0.327±0.041^b^	0.311 ± 0.009^bc^
5.	40 CBs	0.25±0.038^c^	0.205 ± 0.019^c^

*X ±* S for three replicate measurements at a 95% level of confidence. Different letters indicate a significant difference (*P ≤* 0.05)

### Chlorophyll a fluorescence (ChlF) kinetics

**Fig. 3:**
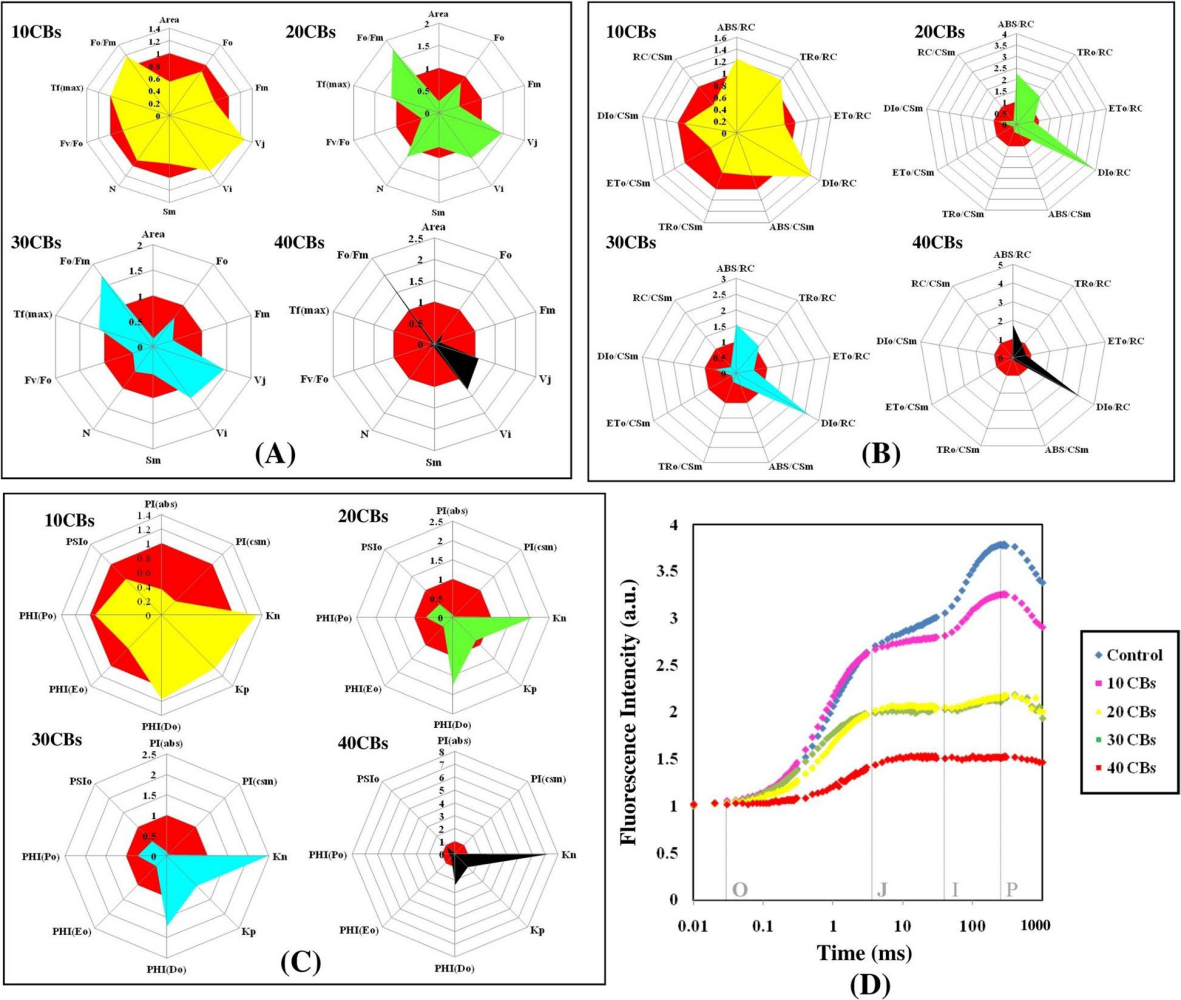
The technical fluorescence parameters were represented by radar plots (**A**-**C**), with each line showing the average of 6 measurements per treatment and statistical significance was determined at *p* ≤ 0.05 levels. Plot (**D**) The study measured ChlF in *A. pinnata* plants exposed to varying concentrations of CBs (0-40) for 24 hours, using PSII rapid fluorescence transients (O, J, I, and P) as indicators

ChlF of *A. pinnata* was measured after 24 h of CBs treatment and a typical OJIP induction curve was displayed when plotted on the logarithm time scale in Fig. 3D. With increasing the CBs concentration, the fluorescence yield at various intermediary steps, such as J, I, and P was reduced. In control plants, two intermediate peaks F_J_ (chlorophyll fluorescence at 2 ms) and F_I_ (chlorophyll fluorescence at 300 ms) were formed between F_0_ and F_M_, ChlF increased continuously from F_0_ to F_M_ fluorescence intensity in *A. pinnata* growing under control conditions. CBs induced reduction in PSII photochemistry and electron transport activity were severe at the highest concentration of CBs.

### Biophysical parameters

**Fig. 4:**
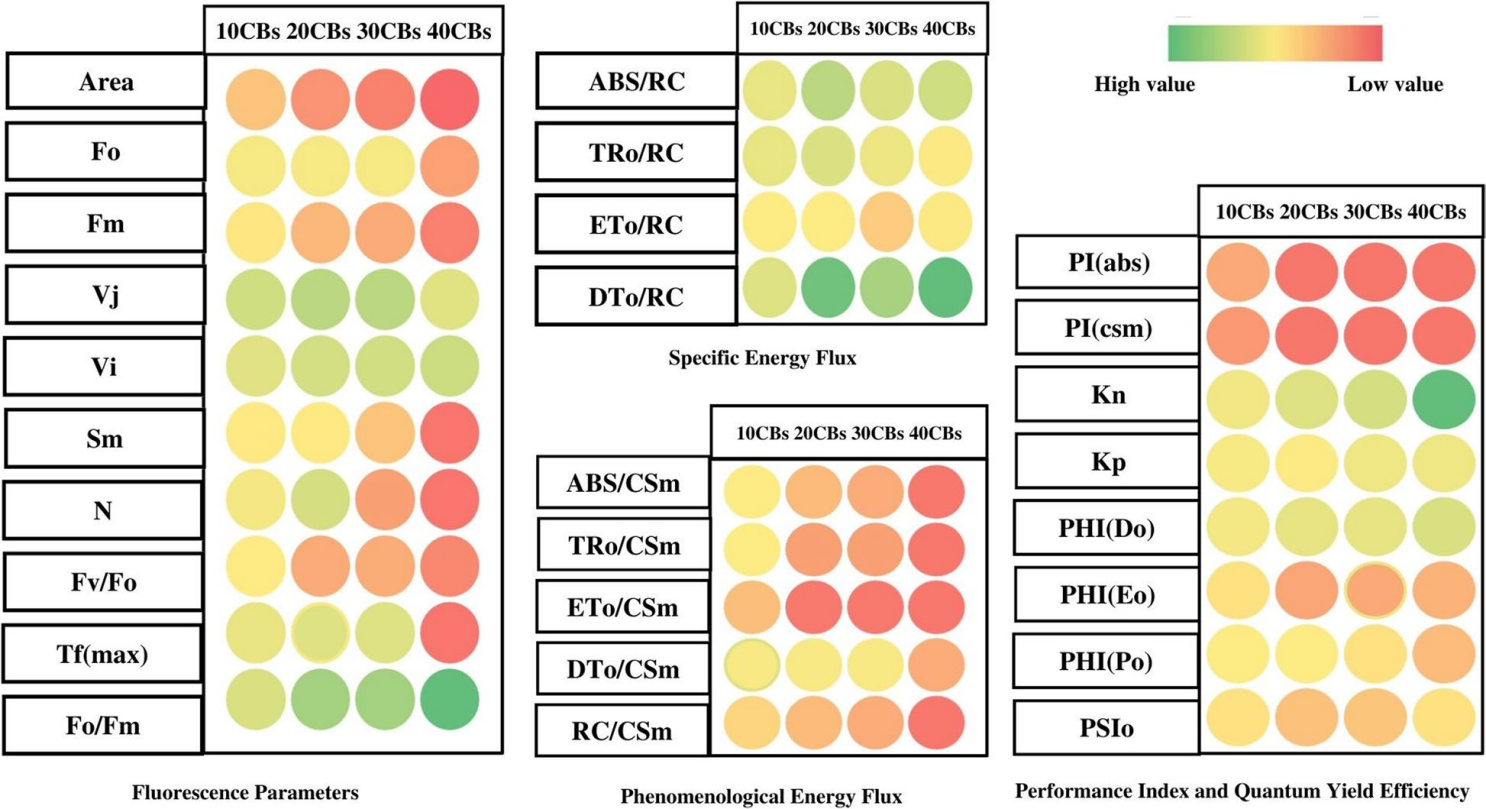
A heat map was used to illustrate the relative variability of multiple photosynthesis-related parameters obtained from the JIP test on *A. pinnata* plants under CBs stress. The data was collected for varying concentrations (0-40) after 24 hours, with red indicating lower values (1%), yellow indicating medium (50%), and green indicating the highest values (100%). Prior to color coding, all data was normalized to maintain unbiased results within a range of 1-100 for the parameter values

The concentration of CBs has been found to decrease both the smallest fluorescence intensity (F_0_) and the maximum fluorescence intensity (F_M_), as shown in Figs. 3A & 4. F_0_ represents the fluorescence intensity measured at 50 μs when the primary (1°) quinone acceptor (Q_A_) is in the oxidized state. The effectiveness of photosynthesis in plants is closely related to the maximum 1° yield of photochemistry of PSI1, which is reflected by the Fv/F_0_ ratio supposed to be relation of the rates at which excited Chl pigment undergo photochemical and non-photochemical deactivation. Raising value of F_v_/F_0_ indicates proper performance of PSII. However, the F_v_/F_0_ ratio for *A. pinnata* plants decreased steadily with increasing CBs concentration, as shown in Figs. 3A & 4. At 10 CBs, the F_v_/F_0_ ratio was 79.13 % of control, while at 20 CBs; it decreased to 40.55 % of control. Similarly, at 30 CBs and 40 CBs, the F_v_/F_0_ ratio was found to be 41.73 % and 19.29 % of control, respectively.

V_J_, is the relative variable fluorescence at 2 ms (J step) which measures the 1° quinone e^-^ acceptor of PSII in its reduced state [Q_A_^-^/Q_A_ (total)] [41]. V_J_ was found to increase as the concentration of CBs amplified. The maximum raise observed was up to 149 % of control value at 30 CBs, as depicted in Figs. 3A & 4.

The Complimentary Area (S_M_) is a crucial factor that is directly linked to the number of redox events (reduction and oxidation) of one Q_A_ molecule throughout the rapid OJIP transient, in short the number of e^-^ that pass through the electron transport chain (ETC) [40]. The Turnover Number (N) refers to the number of times that Q_A_ is reduced and re-oxidized until Fm is attained [44–46]. In *A. pinnata*, both S_M_ and N values decrease with an increase in CBs concentration (Figs. 3A & 4). Specifically, S_M_ decreases by up to 8.7 % from the control, while N decreases by up to 7.1 % of control at the highest CBs concentration.

### Quantum yield

The introduction of CBs in plants led to a slight reduction in the quantum yield of 1° photochemistry (φP_0_) and electron transport (φE_0_), which are indicators of the complete photosynthetic efficiency of active PSII reaction centre (RC). This trend was evident in both Figs. 3C and 4. The minimal values of φP_0_ and φE_0_, which were approximately half of control, were recorded when *A. pinnata* was exposed to 40 CBs. In contrary, the quantum yield of dissipitation (φD_0_) showed a continuous enhancement with increasing CBs concentration, with approximately a two-fold increment observed from the control in the 40 CBs condition.

### Specific energy flux (membrane model)

**Fig. 5:**
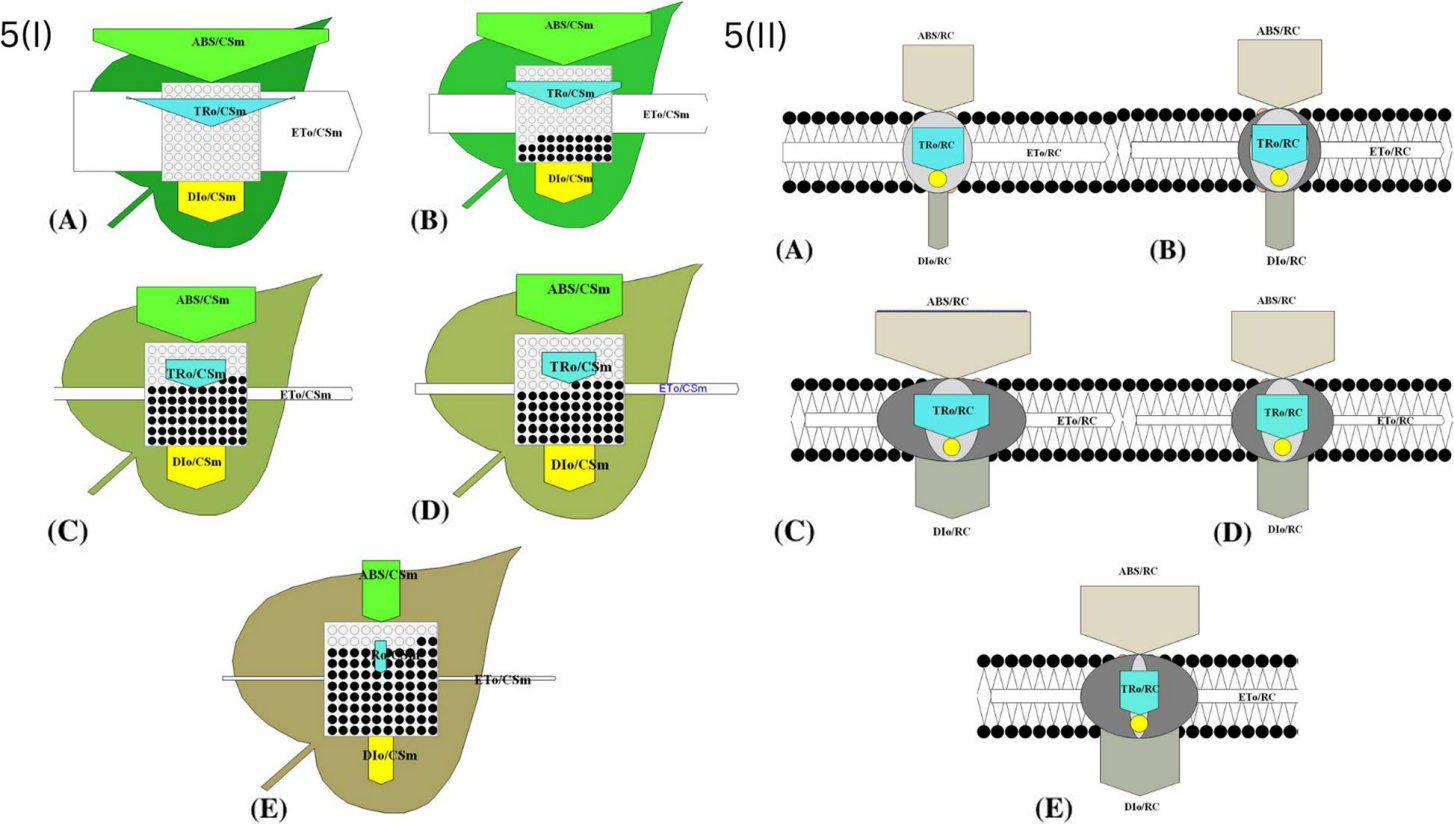
(I): The study utilized an energy pipeline leaf model to investigate the phenomenological fluxes (per cross section, CS) in *A. pinnata* fronds under different concentrations of CBs, (**A**); control, (**B**); 10 CBs (**C**); 20 CBs (**D**); 30 CBs and (**E**); 40 CBs. (II): The study employed a thylakoid membrane model to analyze the specific energy fluxes (per reaction, RC) in *A. pinnata* fronds exposed to various concentrations of CBs, (**A**); control, (**B**); 10 CBs (**C**); 20 CBs (**D**); 30 CBs and (**E**); 40 CBs

The study analyzed the photosynthetic performance of active PSII RC of *A. pinnata* under differential concentrations of CBs by examining specific energy fluxes such as absorption energy (ABS/RC), trapped energy (TR_0_/RC), electron transport (ET_0_/RC), and dissipated energy (DI_0_/RC) flux per reaction centre, showing in Figs. 3B & 5(II). The results showed a significant increase in ABS/RC and TR_0_/RC at 40 CBs condition, indicating an enhancement in the absorption potential of active RC (Figs. 3B, 4). TR_0_/RC was highly raised at 30 CBs condition that is 110.75 % of control. When exposed to increasing concentrations of CBs, the plants showed a decrease in ET_0_/RC, and at higher treated conditions, it reached 72.72 % of the control. On the other hand, the DI_0_/RC displayed a significant increase in a sequential manner, with around a fivefold increment noted in plants treated with 40 CBs in comparison to the control. The study utilized thylakoid membrane models to diagrammatically present the effects of CBs on specific energy fluxes 5(II). Moreover, the study investigated whether CBs alters the ABS to active PSII RC ratio. The findings indicated that severe CBs stress results in more inactive RC and reduced ability of RC to reduce plastoquinone, as reflected by higher values of specific energy fluxes (ABS/RC, TR_0_/RC, and DI_0_/RC) in the leaf pipeline model.

### Phenomenological energy flux (leaf model)

The impact of CBs-induced stress on *A. pinnata* was observed through changes in phenomenological energy fluxes such as absorption (ABS/CSm), trapped energy (TR/CSm), electron transport (ET/CSm), and dissipated energy (DI/CSm) flux per cross section. All these parameters showed significant reduction with increasing CBs concentration in *A. pinnata*. Specifically, at the 40 CBs condition, ABS/CSm, TR/CSm, ET/CSm, and DI/CSm decreased by 0.13 %, 6 %, 27.15 %, and 32.90 %, respectively, compared to the control Fig. 5 (I).

### K_P_ and K_N_

Under CBs stress, the rate constants for non-photochemical de-excitation reactions (K_N_) were found to increase, and at severe stress levels, the K_N_ value reached up to 741.66 % of control, as depicted in Figs. 3C and 4. On the other hand, the de-excitation rate constants for photochemical reactions (K_P_) only showed a slight increase under all stress conditions.

### Performance Index

**Fig. 6:**
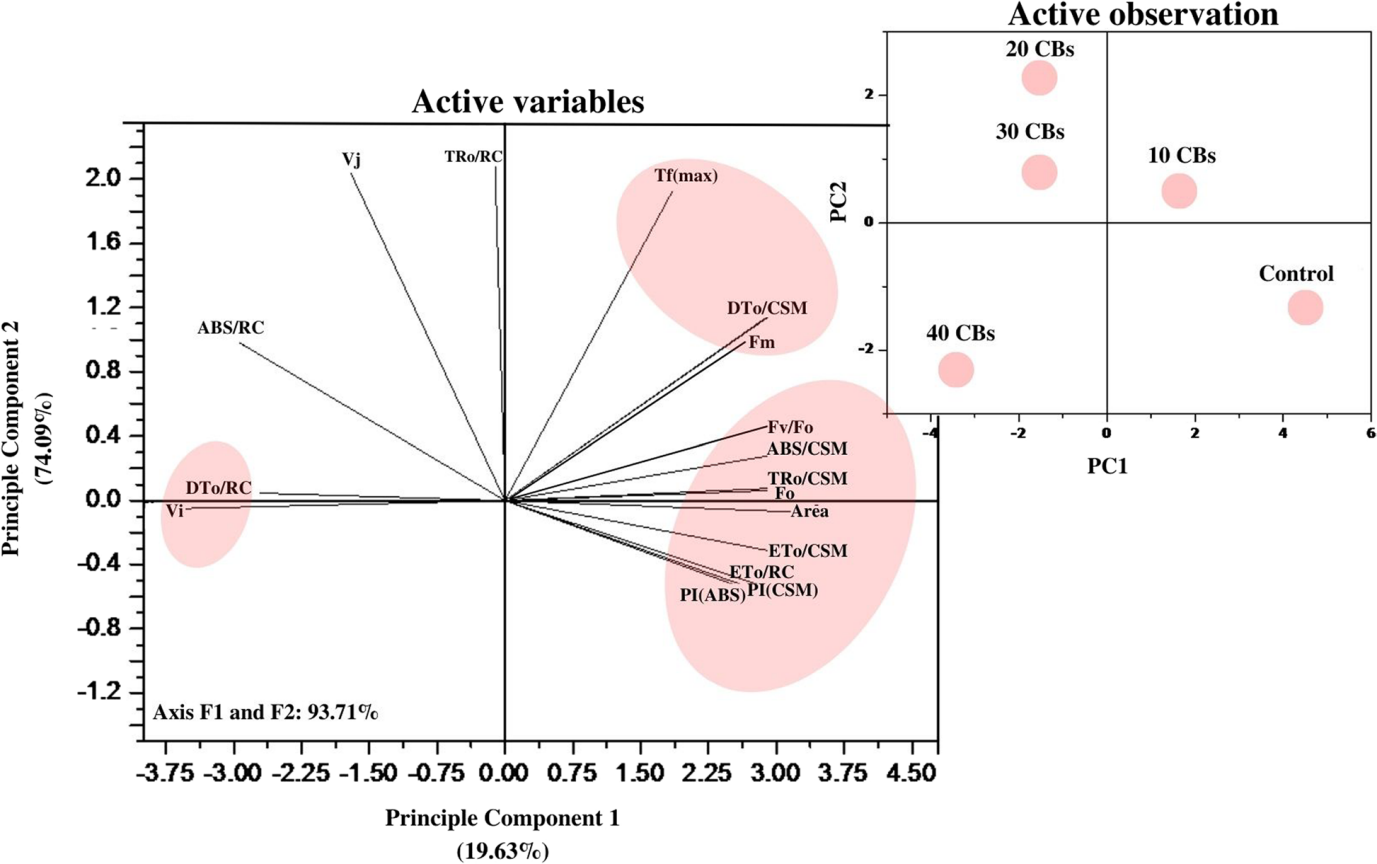
A Principal Component Analysis (PCA) was conducted using chlorophyll fluorescence data for four different CBs treatment conditions. The PCA generated two dimensions (PC1 and PC2), with PC2 capturing the majority of the variance in the data. The Chlorophyll a fluorescence parameter was represented by arrows on the PC1 and PC2 dimensions. All calculated chlorophyll a fluorescence parameters. The correlations were represented with a color code.

**Fig. 7:**
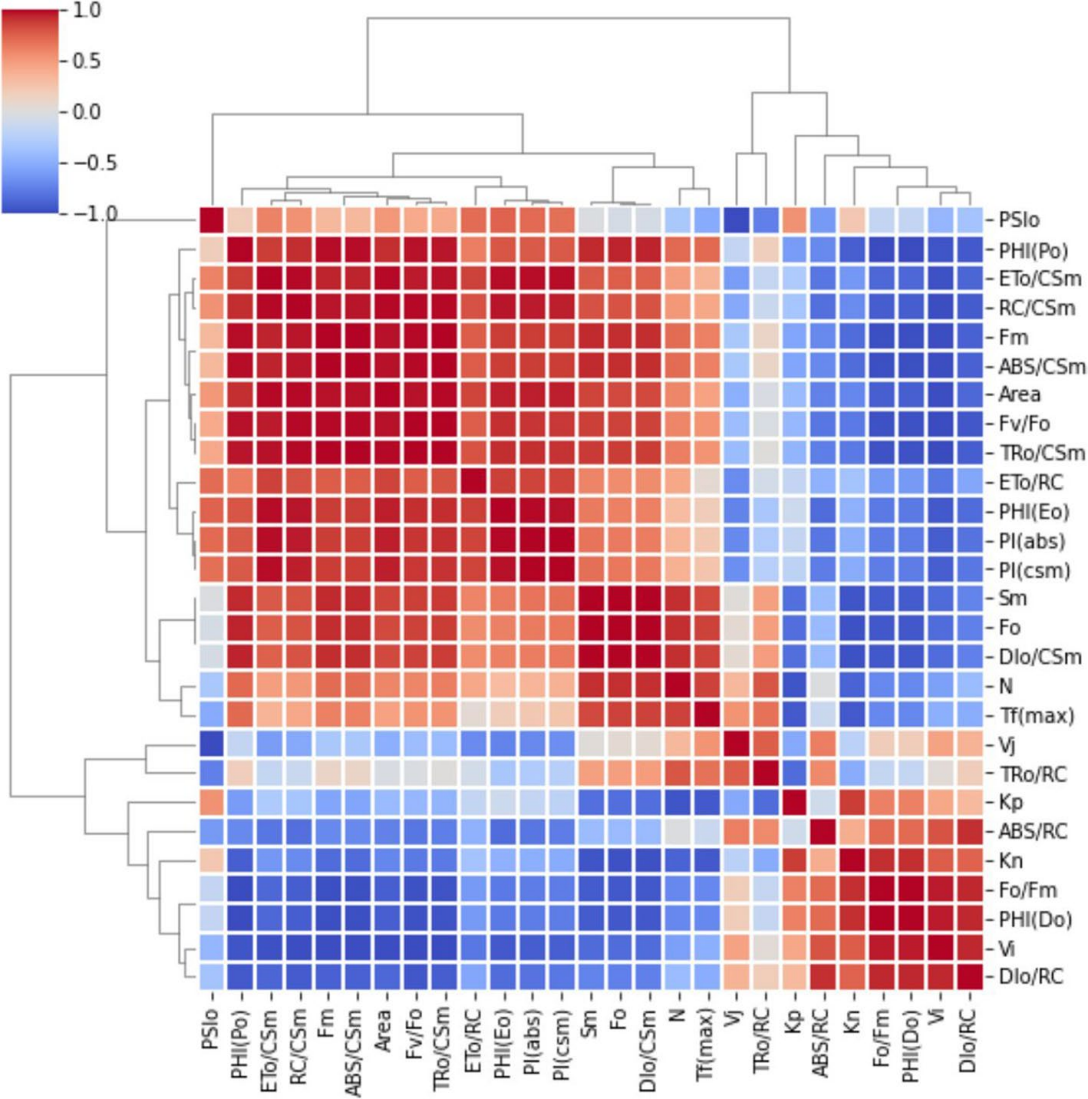
Grid correlation matrix shows the correlation between all calculated chlorophyll *a* fluorescence parameter (with color code)

A radar plot (Figs. 3, 4) was used to illustrate the overall impact of CBs-induced stress on various photosynthetic parameters. In order to assess the effects of CBs on the overall performance of photosynthesis, PI_ABS_ (performance index on absorption basis) and PI_CS_ (performance index of PSII and PSI) were measured in *A. pinnata* plants subjected to different intensities of CBs stress. The results showed that CBs had a significant effect on PI_ABS_ and PI_CS_, with both parameters decreasing continuously as the concentration of CBs increased. The lowest values of PI_ABS_ and PI_CS_ were recorded, which were respectively 11 times and 80 times lower than the control (as shown in Figs. 3C and 4). The results of the PCA analysis showed that the first two principal components, Dim 1 and Dim 2, explain 93.71% of the total variation in the ChlF parameter under CBs induced stress in *A. pinnata* (Fig. 6). The loadings for several JIP parameters are located in quadrant I and IV, including ET_0_/RC, ET_0_/CSm, PI_CS_, PI_ABS_, ABS/RC, TR_0_/RC, and DT_0_/RC. Meanwhile, TR_0_/CSm, ABS/CSm, DI_0_/CSm, F_0_, F_M_, and F_v_/F_0_ are accounted for in quadrant II. Most treatments, except for the 40 CBs treatment, are found in quadrant II and IV. However, the 40 CBs treatment has a longer loading arrow than others in all quadrants, indicating that it significantly affects the major JIP parameters located in quadrant I and III (Fig. 6). Along this correlation matrix has been showing for all parameters in Fig. 7.

The LD_50_ and LD_90_ values for *A. pinnata*, which were calculated by number of mortality rate through probit analysis with a 95 % probability level displays in Table 4. LD_50_ and LD_90_ represent the lethal dose necessary to cause 50 % and 90 % mortality, respectively. The values obtained for LD_50_ is 20.30 CBs and LD_90_ is 35.26 CBs.

**Table 4:** The probit analysis was used to determine the acute 48-hour LD_50_ values of CBs in *A. pinnata*, along with their corresponding confidence limits. The logarithm used in the analysis was base 10

	**Confidence Limits**						
	Probability	95% Confidence Limits for treatment			95% Confidence Limits for log(treatment)^a^		
		Estimate	Lower Bound	Upper Bound	Estimate	Lower Bound	Upper Bound
PROBIT	.010	7.453	5.243	9.347	.872	.720	.971
	.020	8.381	6.096	10.303	.923	.785	1.013
	.030	9.030	6.706	10.962	.956	.826	1.040
	.040	9.551	7.204	11.486	.980	.858	1.060
	.050	9.996	7.635	11.933	1.000	.883	1.077
	.060	10.392	8.022	12.328	1.017	.904	1.091
	.070	10.751	8.376	12.685	1.031	.923	1.103
	.080	11.084	8.706	13.015	1.045	.940	1.114
	.090	11.395	9.018	13.323	1.057	.955	1.125
	.100	11.690	9.313	13.614	1.068	.969	1.134
	.150	12.992	10.638	14.897	1.114	1.027	1.173
	.200	14.129	11.813	16.016	1.150	1.072	1.205
	.250	15.184	12.912	17.059	1.181	1.111	1.232
	.300	16.199	13.974	18.069	1.209	1.145	1.257
	.350	17.199	15.020	19.078	1.236	1.177	1.281
	.400	18.205	16.068	20.109	1.260	1.206	1.303
	.450	19.235	17.131	21.185	1.284	1.234	1.326
	.500	20.305	18.220	22.331	1.308	1.261	1.349
	.550	21.434	19.348	23.576	1.331	1.287	1.372
	.600	22.647	20.530	24.956	1.355	1.312	1.397
	.650	23.972	21.786	26.518	1.380	1.338	1.424
	.700	25.452	23.145	28.327	1.406	1.364	1.452
	.750	27.152	24.653	30.485	1.434	1.392	1.484
	.800	29.179	26.388	33.158	1.465	1.421	1.521
	.850	31.734	28.495	36.661	1.502	1.455	1.564
	.900	35.269	31.300	41.713	1.547	1.496	1.620
	.910	36.180	32.007	43.049	1.558	1.505	1.634
	.920	37.197	32.789	44.555	1.571	1.516	1.649
	.930	38.347	33.666	46.278	1.584	1.527	1.665
	.940	39.675	34.668	48.289	1.599	1.540	1.684
	.950	41.245	35.841	50.698	1.615	1.554	1.705
	.960	43.169	37.262	53.693	1.635	1.571	1.730
	.970	45.658	39.076	57.634	1.660	1.592	1.761
	.980	49.190	41.610	63.347	1.692	1.619	1.802
	.990	55.320	45.912	73.572	1.743	1.662	1.867

^a^Logarithm base = 10

## Discussion

Numerous studies have investigated the physiological adaptations of plants subjected to various stresses, which have revealed that plants have developed intricate defense mechanisms to counteract the detrimental effects of these environmental stressors [45, 47, 48]. The technique of chlorophyll *a* fluorescence (ChlF) analysis has been widely employed to detect composite biochemical changes occurring in the photosynthetic apparatus of plants, encompassing both terrestrial and aquatic species [49]. The present investigation focused on assessing the impact of CBs exposure on multiple fluorescence parameters of photosystem II in *Azolla pinnata*.

### Morphological parameters

The toxicity of CBs is widely acknowledged to have an adverse impact on the growth and physiological health of aquatic plants, albeit the degree of impact may vary depending on the specific plant species. In this particular study, it was observed that exposure to low concentrations of CBs (10) resulted in a minor reduction in frond area and root length in *A. pinnata* plants. However, as the concentration of CBs increased (≥20 CBs), a significant reduction in frond area and root length was observed. Previous studies also demonstrate that lower concentration produces less effect where as high concentration greater than 20 Earlier results also retarded the plant growth [50]. The notable decline in AFA and ARL at higher CBs concentrations suggests a negative correlation between CBs and plant growth. Furthermore, over time, plant mortality rates increased with CBs treatment, and a decrease in frond number was attributed to excessive exposure to CBs, which also negatively impacted survival.

### Biophysical parameters

The minimal fluorescence intensity values serve as a crucial parameter that can offer valuable information on the irreparable harm caused to PSII, which is connected to the light-harvesting complex II (LHCII), this damage can impede the transfer of e^-^ on the reduced side of PSII [51, 52]. A decrease in minimal fluorescence under high CBs stress could be associated with less efficient PSII activity due to conformational changes in the D1 protein caused by CBs stress, which further result in alterations in the properties of PSII electron acceptors [51].

The F_V_/F_0_ parameter is utilized to determine the highest achievable 1° yield of photochemistry by considering concurrent variations in F_M_ and F_0_. When fronds are exposed to CBs stress, there is a decrease in F_V_/F_0_ values, which signifies alterations in the electron transport rate to the 1° electron acceptors from PSII and a reduction in the quantity and size of the reaction center. Previous studies have also documented a decrease in the F_V_/F_0_ ratio in different plant species due to environmental stress [53, 54]. The elevated level of relative variable fluorescence (V_J_) under CBs treatment suggests that the electron transfer at the donor side of PSII has been affected. The modified unquenchable fluorescence (F_0_) may have disrupted the energy transfer from the antenna complex to the reaction center, which in turn can impact F_V_/F_0_. The PCA analysis reveals a positive correlation between the dissipiation per reaction center (DT_0_/RC) and the relative variable fluorescence V_i_, whereas a negative correlation is observed with F_0_. This correlation is further validated by the correlation matrix (Fig. 7).

The analysis of fluorescence transients, also known as the "JIP test," in photosynthetic organisms subjected to abiotic stress has indicated a significant decline in the value of φP_0_ [55]. This decrease in φP_0_ can be attributed to a reduction in the photochemical efficiency of PSII caused by CBs-induced stress. Specifically, under light conditions, the reduction in the maximum quantum yield of PSII (φP_0_) signifies that CBs stress impedes the redox reaction following Q_A_ and delays electron transport between Q_A_^-^ and Q_B_ [56]. These parameters are crucial for gaining insights into the electron transport activity at the PSII acceptor sites. The findings of the present study suggest that CBs treatment leads to a reduction in electron transport at the PSII acceptor site in *A. pinnata* [57].

The energy pipeline models, such as the membrane and leaf model presented in Fig. 5(I) & (II), have demonstrated that several sites in PSII are sensitive to multiple environmental strain [58, 59]. The results of the present study indicate that the efficiency of trapping of electron and transport of electron from PSII decreases with an increasing concentration of CBs, as active RC is converted into inactive RC (denoted as dark circle in model). This is reflected in the decrease of TR_0_/CSm and ET_0_/CSm values [39, 41, 60]. The ABS/RC ratio is resolute by the total amount of photons captured by Chl molecules all over RC, divided by the total number of active RC [61]. The ratio of active to inactive RC affects this value, with an increase in active RC resulting in an increased ABS/RC ratio. The TR_0_/RC ratio is an indicator of the maximal rate at which an exciton is captured by the RC, resulting in a decrease in the population of the 1° electron acceptor (Q_A_). An increase in this ratio implies a reduction in the amount of Q_A_ that remains reduced. The decrease in the ET_0_/RC ratio signifies a reduced capacity for electron transport in inactive RC to re-oxidize the reduced Q_A_, as more active RC is available. The total dissipation of un-trapped excitation energy from all RC, divided by the number of active RC, gives the DI_0_/RC ratio, which is influenced by the ratios of active to inactive RC. The dissipation can take place via several pathways, including heat, fluorescence, and energy transfer to other systems and the ratio of active to inactive RC affects this dissipation. However, despite the impact of active/inactive RC ratios, the DI_0_/RC ratio is not significantly affected due to the efficient use of energy by active RC [62, 63].

The F_V_/F_M_ ratio is a crucial parameter in the JIP test that reflects the efficiency of 1° light energy conversion in the PSII reaction center. It serves as a stress indicator in many photosynthetic studies [55, 56, 64, 65]. However, this ratio is reliant on the fluorescence levels of F_0_ and F_M_, and any decrease in Fm value can result in a reduction in F_V_/F_M_ ratio with increasing CBs. A novel and more responsive parameter called the Performance Index for measuring photosynthetic efficiency under stress [65, 66]. The performance index is derived from three or four components based on the density of reaction centers, trapping efficiency, and electron transport efficiency, much like the Goldman equation [67]. Food and productivity of plants are highly sensible with the entrapment of electron through the light reaction. Photosynthesis performance enroll the capability in plant to produce energy for growth and development [68, 69]

The performance index of a plant is a sensitive measure of the effects of stress on its components. Performance index is calculated based on energy absorption (PI_ABS_) and cross-section (PI_CS_), with the latter being dependent on the phenomenological energy flux. Our study found that the presence of CBs significantly lowered the values of PI_ABS_ and PI_CS_ in *A. pinnata*. This decrease in PI_ABS_ was attributed to reduced activity of the RC, which ultimately decreased the overall activity of the RC [30, 51, 70]. Using statistical models such as PCA and Correlation matrix, we identified several JIP parameters, including ABS/CSm, TR_0_/CSm, ET_0_/CSm, φP_0_, PI_ABS_ and PI_CS_, which exhibited a dose-response relationship under CBs stress. Furthermore, the LD_50_ values of CBs, obtained through probit analysis, indicated that this molecule is highly toxic to *A. pinnata.*

## Limitation and future aspects

Cigarette litter, particularly the disposal of CBs, is often driven by misconceptions regarding their environmental impact and perceived rapid biodegradability. A significant proportion of smokers, such as 43% surveyed in Germany [35, 71], are unaware that cigarette filters are predominantly composed of synthetic material, specifically cellulose acetate—a type of plastic. Despite this composition, CBs are not widely recognized as single-use plastics. However, emerging evidence from various studies has demonstrated the detrimental effects of CBs on terrestrial, freshwater, and marine ecosystems [2, 7, 21]. The prolonged presence of these filters can have adverse ecological consequences, including diminished growth and biomass of economically significant primary producers, potentially leading to cascading effects on entire ecosystems.

As a result, there is an urgent need to reclassify cellulose acetate CBs globally as single-use plastics. Such a classification is crucial to improve regulations pertaining to their usage, collection, and disposal. To safeguard the environment effectively, a multifaceted approach is required. This includes raising awareness through targeted campaigns that educate the public about the severe impacts of cigarette litter. Furthermore, imposing higher fines and implementing smoking bans in ecologically sensitive areas [72] can act as deterrents. Additionally, extended producer responsibility must be enforced on tobacco companies, compelling them to take accountability for the collection, transportation, processing, and disposal of tobacco product waste. It is evident from interviews conducted by [73] that smokers often do not perceive cigarette filters as litter, highlighting the necessity of fostering public awareness regarding the long-lasting persistence of even biodegradable filters in the environment [74].

## Conclusions

In conclusion, the current study provides compelling evidence of the significant impact of CBs on the morphology, ChlF kinetics and photosynthesis efficiency of *A. pinnata* plants, primarily by modulating the photosynthetic process. The study indicates that the introduction of CBs led to a decrease in chlorophyll fluorescence kinetics, quantum yield, and energy fluxes related to electron transport, while the energy fluxes related to absorption and dissipation increased. These findings suggest that CBs interfere with the photosynthetic process and alter the functioning of the PSII reaction centers, resulting in reduced growth and productivity of *A. pinnata*. The study highlights the need to regulate the use of CBs to mitigate their potential impact on plant growth and productivity. On the basis of lethal dose value (LD_50_ and LD_90_) plants are not able to survive on average 20-30 CBs concentration. Raising awareness regarding the long-lasting effects of cigarette filters, despite their biodegradability, is essential. Such CBs may persist in the environment for extended periods, leading to detrimental ecological consequences. They can curtail the growth and biomass of primary producers with economic significance, resulting in cascading impacts on ecosystems. There is a paucity of knowledge about the negative impacts of discarded CBs on terrestrial and aquatic ecosystems. Thus, it is imperative to acknowledge this concern and take appropriate measures to alleviate the harmful influence of CBs on the environment.

## Ethics approval and consent to participate

I declare that experimental research and field studies on plants (either cultivated or wild), must comply with the relevant institutional, national, and international guidelines of OECD 2002 and legislation. All methods were performed in accordance with the relevant guidelines and regulations.

## Consent for publication

Not applicable.

## Competing interests

The author declare that they have no competing financial interest or personal relationship that

## Data Availability

The data and materials that support the findings of the study are available from the corresponding author upon request.

## Abbreviations

CBs: Cigarette butts
RC: Reaction center
PSI: Photosystem I
PSII: Photosystem II
ChlF: Chlorophyll *a* fluorescence
